# Clinicogenomic Features and Outcomes of Adenoid Cystic Carcinoma With Central Nervous System Metastases: A Single‐Institution Cohort Study

**DOI:** 10.1002/hed.70342

**Published:** 2026-06-08

**Authors:** Omar Elghawy, Jack Stylli, Martin Kurian, Eitan Halper‐Stromberg, Aditi Singh, Christopher D'Avella, Lee Hartner, Roger B. Cohen

**Affiliations:** ^1^ Division of Hematology/Oncology, Department of Medicine Abramson Cancer Center, University of Pennsylvania Philadelphia Pennsylvania USA; ^2^ Department of Pathology and Laboratory Medicine University of Pennsylvania Philadelphia Pennsylvania USA

**Keywords:** adenoid cystic carcinoma, central nervous system metastasis, leptomeningeal disease, next‐generation sequencing, perineural spread, salivary gland cancer

## Abstract

**Background:**

Adenoid cystic carcinoma (ACC) is a rare malignancy with a propensity for perineural invasion and hematogenous spread. Central nervous system (CNS) involvement is uncommon, and detailed clinical and genomic data on this aspect of the disease remain limited.

**Methods:**

We performed a single‐institution retrospective cohort study of patients with biopsy‐confirmed ACC and CNS involvement treated at the University of Pennsylvania. Clinical features, CNS disease patterns, treatment modalities, molecular alterations, and survival outcomes were abstracted from the electronic medical record.

**Results:**

Seventeen patients were identified. CNS involvement occurred a median of 40 months after initial ACC diagnosis. CNS disease included parenchymal metastases, leptomeningeal disease, and patterns suggestive of neurotropic or perineural intracranial extension. Leptomeningeal involvement was present in 6 patients. Targeted next‐generation sequencing demonstrated NOTCH1 alterations in 7 patients, with additional recurrent alterations in CREBBP, BAP1, FGFR2, and PI3K‐pathway genes. CNS‐directed therapy included surgery, stereotactic radiosurgery, hypofractionated radiotherapy, whole‐brain radiotherapy, and conventionally fractionated radiotherapy. Median progression‐free survival was 5.4 months, and median overall survival was 9.4 months after CNS involvement.

**Conclusions:**

CNS involvement in ACC was associated with poor outcomes despite multimodal therapy. NOTCH1 alterations and leptomeningeal disease were frequent in this cohort, but these findings should be interpreted as hypothesis‐generating given the small sample size and absence of a matched non‐CNS comparator cohort.

## 1 Introduction

Adenoid cystic carcinoma (ACC) is a rare epithelial malignancy accounting for approximately 1% of all head and neck cancers and up to 10% of salivary gland tumors [1, 2]. Although ACC often follows an indolent clinical course, it is characterized by a strong propensity for perineural invasion, local recurrence, and hematogenous spread, most commonly to the lungs [2, 3]. Distant metastases develop in approximately 35% to 50% of patients and may occur many years after initial diagnosis [3].

The therapeutic management of ACC remains challenging. Complete surgical resection is the mainstay of localized treatment, but negative margins may be difficult to achieve because of perineural extension and anatomically complex head and neck primary sites [4]. Postoperative radiation is commonly used, although its survival benefit remains debated [5, 6, 7]. No systemic therapies are currently approved specifically for ACC. Cytotoxic chemotherapy regimens, including cyclophosphamide, doxorubicin, and cisplatin, have produced modest response rates in recurrent or metastatic disease; multitargeted kinase inhibitors such as lenvatinib, apatinib, and axitinib may produce disease stabilization in some patients [8, 9, 10, 11]. Overall, durable objective responses remain uncommon [12, 13].

Central nervous system involvement from ACC is rare and has been described mostly in case reports and very small series [14, 15, 16]. CNS disease in ACC is also biologically and anatomically heterogeneous. Some patients develop predominantly parenchymal brain lesions suggestive of hematogenous dissemination, whereas others develop intracranial involvement through skull base, dural, cranial nerve, or leptomeningeal patterns suggestive of neurotropic or perineural spread [4, 17, 18]. Leptomeningeal involvement may arise in the setting of disseminated metastatic disease or via direct neurotropic extension and is associated with substantial neurologic morbidity.

Genomic studies have identified molecularly distinct ACC subgroups, including tumors with activating NOTCH1 alterations, which have been associated with aggressive clinicopathologic behavior and inferior survival [19, 20, 21, 22, 23, 24]. Whether NOTCH1‐mutant ACC is specifically predisposed to CNS involvement remains uncertain. In this study, we describe the clinical characteristics, CNS disease patterns, molecular features, treatment modalities, and outcomes of 17 patients with ACC and CNS involvement treated at a single institution.

## 2 Methods

### 2.1 Study Design and Patient Identification

We conducted a retrospective cohort study of patients with biopsy‐confirmed ACC and CNS involvement treated at the University of Pennsylvania between December 1, 2009, and December 31, 2024. This study was approved by the University of Pennsylvania Institutional Review Board and was conducted in accordance with institutional and ethical standards. Patients were identified through an institutional cancer registry query and manual review of the electronic medical record.

CNS involvement was defined by radiographic evidence on MRI or CT, histologic confirmation of ACC from a CNS lesion, or cerebrospinal fluid cytology consistent with malignant involvement by ACC. Clinical, treatment, radiographic, and molecular data were abstracted from the electronic medical record. Molecular profiling was performed using the Penn Medicine Solid Tumor Next‐Generation Sequencing panel, FoundationOne testing, or other clinically available platforms.

### 2.2 Classification of CNS Involvement

To address the heterogeneity of CNS disease in ACC, CNS involvement was categorized by dominant radiographic pattern. Hematogenous parenchymal metastasis was defined as one or more discrete intra‐axial brain lesions without clear contiguous skull base, dural, or cranial nerve extension. Leptomeningeal disease was defined by radiographic leptomeningeal enhancement, cerebrospinal fluid cytology, or clinical documentation consistent with leptomeningeal involvement. Neurotropic or perineural intracranial extension was defined by involvement of the skull base, cavernous sinus, cranial nerves, foramina, dura, brainstem‐adjacent structures, or contiguous extension from a head and neck primary site [4, 17, 18]. Cases with overlapping features or insufficient imaging detail were classified as mixed or indeterminate.

Patients were also categorized as having *de novo* CNS involvement if CNS disease was present at or near the time of initial ACC diagnosis, or metachronous CNS involvement if CNS disease developed during subsequent follow‐up.

### 2.3 Treatment and Response Assessment

CNS‐directed treatment modalities were abstracted, including surgical resection, stereotactic radiosurgery, Gamma Knife radiosurgery, hypofractionated stereotactic radiotherapy, conventionally fractionated radiotherapy, whole‐brain radiotherapy, and systemic therapy administered after CNS diagnosis. Systemic therapy was categorized as cytotoxic chemotherapy, targeted therapy, immunotherapy, hormonal therapy, or investigational therapy.

Radiographic response was abstracted from clinical radiology reports and treating oncologist documentation. When available, intracranial and extracranial responses were recorded separately. Best documented response was categorized as partial response, stable disease, progressive disease, or non‐evaluable/unknown. Patients without measurable disease or adequate follow‐up imaging were classified as non‐evaluable rather than imputed as nonresponders.

### 2.4 Statistical Analysis

Descriptive statistics were used to summarize baseline characteristics, CNS disease patterns, treatment modalities, molecular findings, and outcomes. Continuous variables were summarized as medians and ranges; categorical variables were summarized as counts and percentages. Progression‐free survival and overall survival were estimated using Kaplan–Meier methods from the date of CNS involvement. Patients were censored at last follow‐up.

Given the small sample size, all statistical analyses were considered exploratory and hypothesis‐generating. Comparisons by ECOG performance status, leptomeningeal involvement, NOTCH1 alteration status, and CNS disease pattern were performed descriptively. Formal hypothesis testing and multivariable modeling were limited by the size and heterogeneity of the cohort and were not used to establish independent predictors of outcome. Analyses and figure generation were performed using R version 4.3.0.

## 3 Results

### 3.1 Patient Characteristics

Seventeen patients with ACC and CNS involvement were identified. Baseline characteristics are summarized in Table 1. The median age at initial ACC diagnosis was 54 years, with a range of 34–75 years. Ten patients were female and seven were male. Most patients were White, and ECOG performance status was 0–1 in 11 patients. Primary tumors most commonly arose in the head and neck, including the parotid gland, submandibular gland, and minor salivary glands. The lungs were the most common extracranial metastatic site. Stage IV disease was present at initial diagnosis in 10 patients.

**TABLE 1:** Cohort characteristics (expanded; with counts and percentages).

Characteristic	Value
Total patients	17
Median age at initial ACC diagnosis	54 years (range 34–75)
Sex	Female: 10 (58.8%); Male: 7 (41.2%)
Race	White: 13 (76.5%)
ECOG performance status	0–1: 11 (64.7%)
Primary site (overall)	Head and neck: 13 (76.5%)
Primary site (breakdown within head and neck)	Parotid: 5 (29.4%); Submandibular: 3 (17.6%); Minor salivary glands: 5 (29.4%)
Most common extracranial metastatic site	Lungs: 12 (70.6%)
Stage at initial diagnosis	Stage IV: 10 (58.8%)
CNS disease burden	Multiple CNS lesions: 11 (64.7%)
Leptomeningeal involvement	6 (35.3%)
Neurologic symptoms at time of CNS involvement	Headache: 5 (29.4%); Vision changes: 3 (17.6%); Cranial nerve palsies: 2 (11.8%)

*Note:* Baseline demographic, clinical, primary tumor, extracranial metastatic, CNS disease burden, leptomeningeal involvement, and presenting neurologic symptom characteristics are shown.

Eleven patients had multiple CNS lesions, and 6 patients had leptomeningeal involvement. Neurologic symptoms at the time of CNS involvement included headache, visual changes, cranial nerve palsies, seizure, weakness, behavioral changes, nausea or vomiting, and speech disturbance. Some patients had predominantly parenchymal lesions, whereas others demonstrated skull base, cranial nerve, dural, brainstem, or leptomeningeal involvement suggestive of neurotropic or perineural intracranial extension [4, 17, 18]. These presentations likely represent biologically and clinically distinct patterns of CNS involvement, with differing implications for prognosis and local treatment feasibility.

CNS involvement was present at or near initial ACC diagnosis in 2 patients and developed metachronously during follow‐up in 15 patients. Median time from initial ACC diagnosis to CNS involvement was 40 months. In metachronous cases, the interval from initial diagnosis to CNS involvement varied substantially, consistent with the known tendency of ACC to recur or metastasize after prolonged latency [3, 25]. Patient‐level CNS disease patterns, leptomeningeal status, CNS‐directed treatment modalities, systemic therapy after CNS diagnosis, and best documented systemic responses are summarized in Table 2.

**TABLE 2:** Patient‐level CNS disease pattern and treatment characteristics.

Patient	CNS timing	Radiographic CNS pattern	Leptomeningeal disease	CNS‐directed treatment	Systemic therapy after CNS diagnosis	Best documented systemic response
Patient 1	Metachronous	Parenchymal	No	None documented	Hospice	PD
Patient 2	De novo/near diagnosis	Parenchymal + LMD	Yes	IMRT (10 fractions)	Vinorelbine	PD
Patient 3	Metachronous	Parenchymal + LMD	Yes	Gamma Knife radiosurgery (3 fractions)	Vinorelbine	PD
Patient 4	Metachronous	Parenchymal + LMD	Yes	Whole‐brain radiotherapy (5 fractions)	Mitoxantrone	PD
Patient 5	Metachronous	Parenchymal	No	Gamma Knife radiosurgery (5 fractions); resection	None documented	PD
Patient 6	Metachronous	Indeterminate	No	Gamma Knife radiosurgery (5 fractions)	Lenvatinib	PD
Patient 7	Metachronous	Parenchymal + LMD	Yes	None documented	Trial	NE
Patient 8	De novo/near diagnosis	Parenchymal	No	Gamma Knife radiosurgery (5 fractions)	Cisplatin, vinorelbine; Taselsib	PR
Patient 9	Metachronous	Parenchymal	No	IMRT (5 fractions); resection	None documented	NE
Patient 10	De novo/near diagnosis	Parenchymal	No	IMRT (37 fractions); resection	Lenvatinib; Pembrolizumab	SD
Patient 11	Metachronous	Parenchymal + LMD	Yes	Gamma Knife radiosurgery (10 fractions)	Vinorelbine	PD
Patient 12	Metachronous	Skull base/perineural/mixed	No	None documented	Pembrolizumab	SD
Patient 13	Metachronous	Parenchymal	No	Gamma Knife radiosurgery (several courses); resection	Tamoxifen; CART	PD
Patient 14	Metachronous	Parenchymal + LMD	Yes	Hypofractionated stereotactic radiotherapy (5 fractions)	Axitinib	SD
Patient 15	Metachronous	Parenchymal	No	Whole‐brain radiotherapy (5 fractions)	Bevacizumab	SD
Patient 16	Metachronous	Skull base/perineural/mixed	No	Gamma Knife radiosurgery	None documented	NE
Patient 17	Indeterminate	Parenchymal	No	None documented	Vinorelbine; Mitoxantrone	PR

*Note:* Patient‐level descriptive classification of CNS involvement according to predominant radiographic pattern, including parenchymal disease, parenchymal disease with leptomeningeal involvement, skull base/perineural or mixed patterns, and indeterminate disease. CNS‐directed treatment modalities included surgical resection, stereotactic radiosurgery or Gamma Knife radiosurgery, hypofractionated stereotactic radiotherapy, conventionally fractionated IMRT, whole‐brain radiotherapy, and systemic therapy after CNS diagnosis.

### 3.2 Molecular Features

Molecular profiling by targeted next‐generation sequencing revealed recurrent alterations in NOTCH1, CREBBP, BAP1, FGFR2, and PI3K‐pathway genes, including PIK3CA, PIK3R1, and PIK3C2B (Figures 1 and S1). NOTCH1 alterations were identified in 7 of 17 patients. This proportion is numerically higher than rates reported in several unselected or recurrent/metastatic ACC cohorts, but this comparison is indirect and should not be interpreted as evidence of definitive enrichment in the absence of a matched non‐CNS institutional control group [19, 20, 21, 22, 23, 24].

**FIGURE 1:**
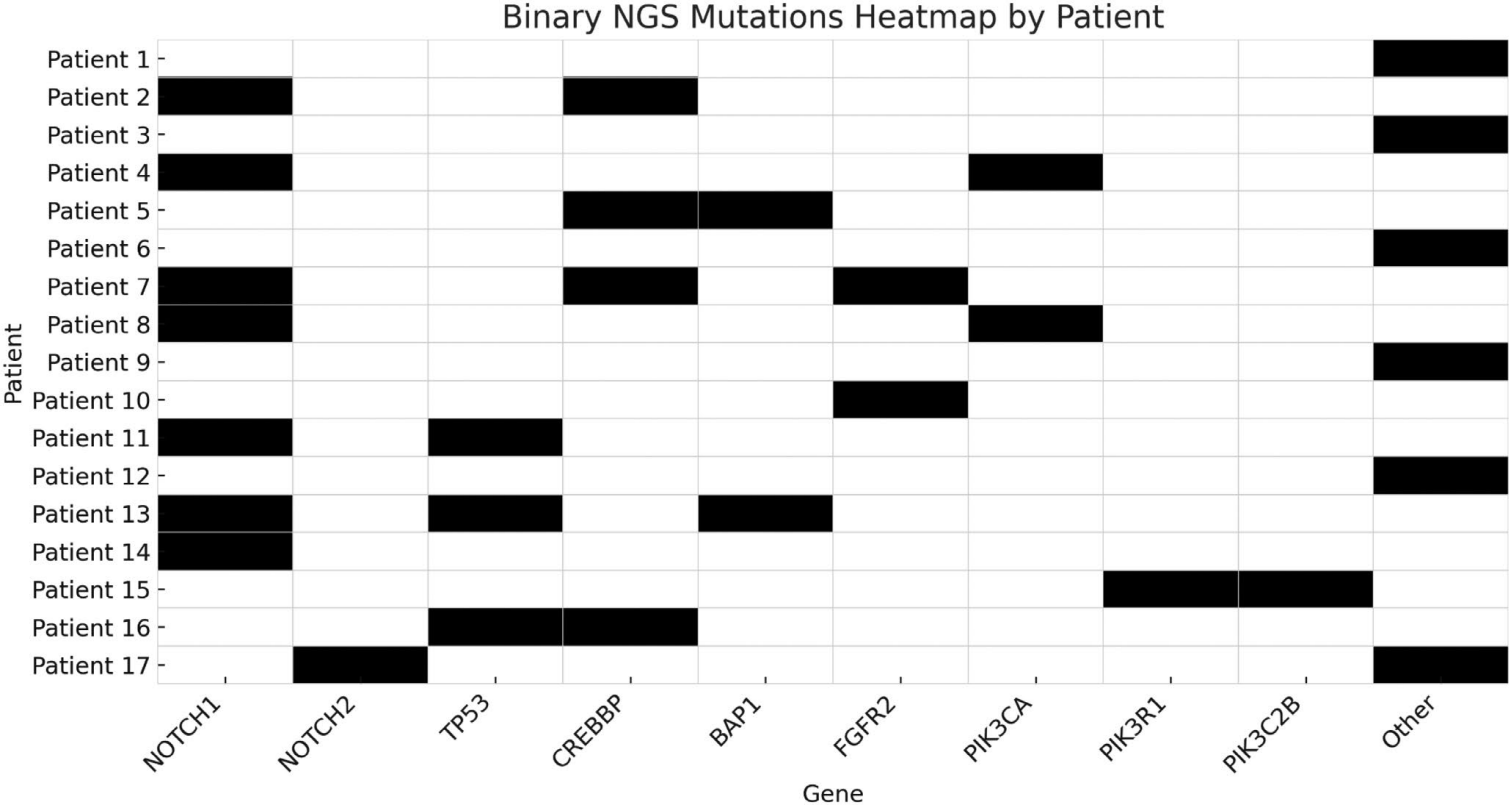
Next‐generation sequencing mutation heatmap. Heatmap of recurrent pathogenic or likely pathogenic molecular alterations identified by next‐generation sequencing. Genes shown include NOTCH1, NOTCH2, TP53, CREBBP, BAP1, FGFR2, PIK3CA, PIK3R1, PIK3C2B, and other recurrent or clinically relevant alterations. Other pathogenic alterations include SLIT2, KMT2C, PTPN11, RASA1, SDHC, CDKN2A, CDKN2B, PTCH1, KDM6A, MTAP, and additional less frequent alterations.

No patient had a molecular alteration directly targetable with an approved therapy. KIT overexpression by immunohistochemistry was present in 8 of 9 tested patients. HER2 testing revealed no actionable alterations among tested patients, and androgen receptor, PSMA, and PD‐L1 testing did not identify actionable expression patterns in the tested subset.

### 3.3 CNS‐Directed Therapy and Systemic Treatment

All patients received multimodal therapy during their disease course. CNS‐directed therapy included stereotactic radiosurgery or Gamma Knife radiosurgery, hypofractionated stereotactic radiotherapy, conventionally fractionated IMRT, whole‐brain radiotherapy, surgical resection, or combinations of these approaches. Treatment selection varied according to lesion number, lesion location, presence of leptomeningeal disease, performance status, neurologic symptoms, prior radiation exposure, and extracranial disease burden.

Fourteen patients received systemic therapy after CNS involvement, including cytotoxic chemotherapy, targeted therapy, immunotherapy, hormonal therapy, and investigational therapy. Systemic treatment was heterogeneous and administered at different points relative to CNS involvement; when possible, response was classified by treatment type and by intracranial versus extracranial disease compartment.

Best documented systemic therapy response was partial response in 2 patients, stable disease in 4 patients, progressive disease in 8 patients, and non‐evaluable or unknown response in 3 patients; no complete responses were observed. Serial target‐lesion measurements were not uniformly available across the cohort; therefore, formal lesion‐level radiographic response assessment and compartment‐specific intracranial versus extracranial response rates could not be reliably calculated for all patients. Response outcomes are therefore presented as best documented qualitative response categories rather than formal RECIST‐based response rates. Kaplan–Meier survival estimates and patient‐level treatment timelines are shown in Figures 2 and 3, respectively. A qualitative response‐category plot summarizing best documented systemic therapy response is provided in Figure 4.

**FIGURE 2:**
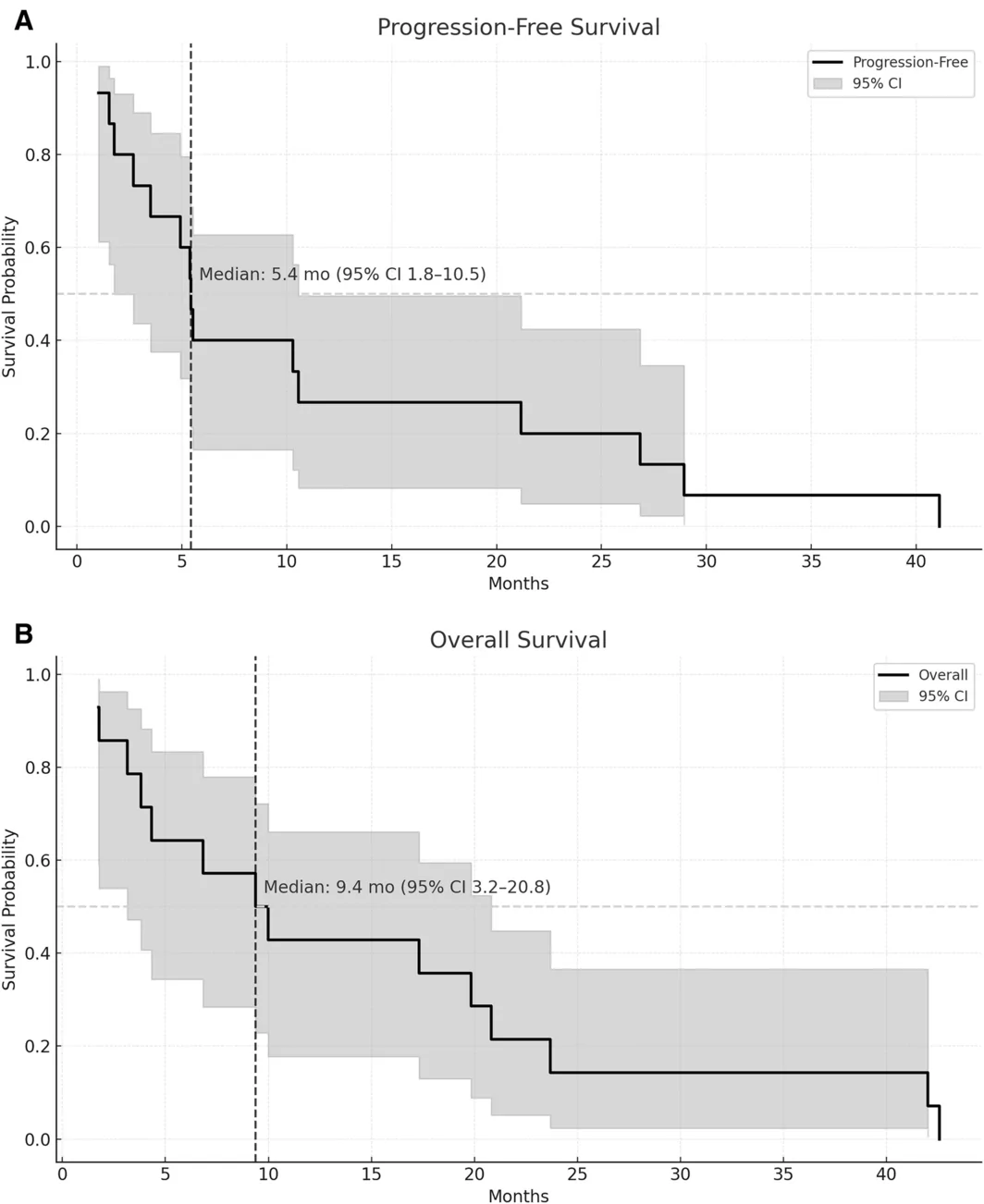
Kaplan–Meier estimates of survival after CNS involvement. Kaplan–Meier curves demonstrating (A) progression‐free survival and (B) overall survival from the date of CNS involvement. Shaded regions represent 95% confidence intervals. Survival estimates should be interpreted as descriptive given the small cohort size.

**FIGURE 3:**
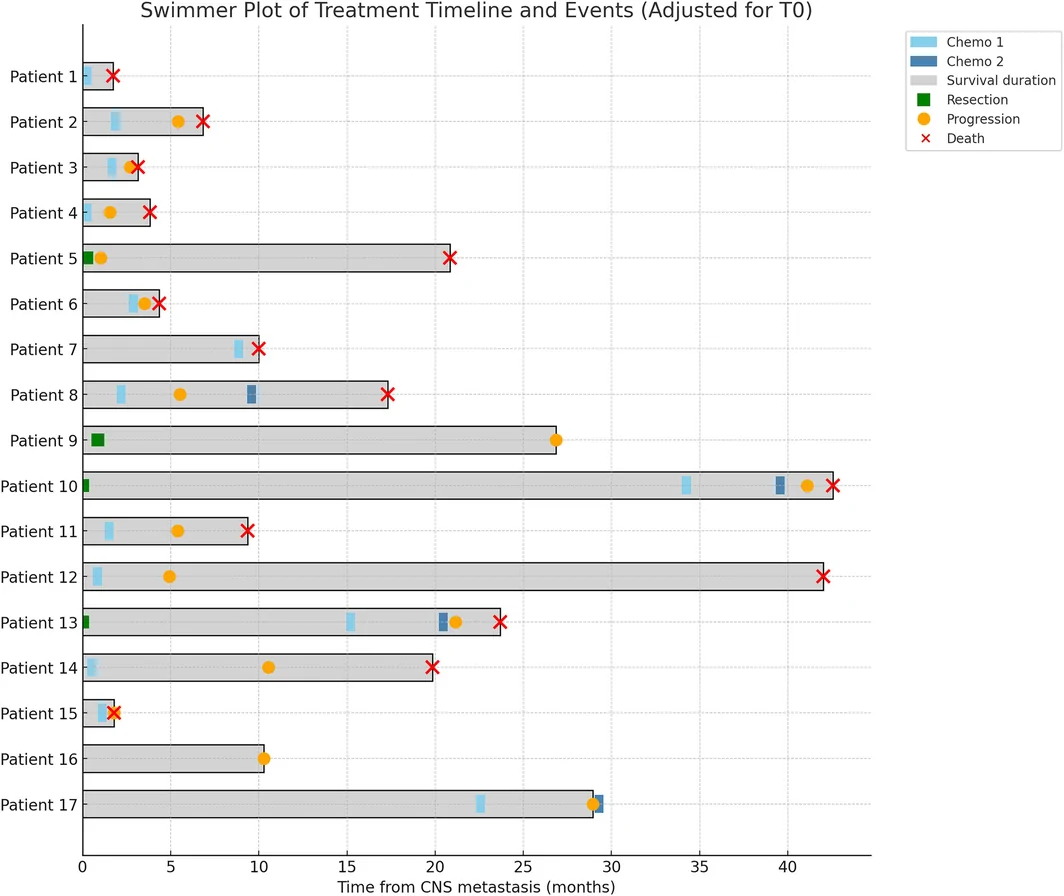
Swimmer plot of treatment timeline and clinical events. Patient‐level swimmer plot showing time from CNS involvement to progression, death or last follow‐up, systemic therapy exposure, CNS‐directed treatment, surgical resection when applicable, progression, and death. [Color figure can be viewed at wileyonlinelibrary.com]

**FIGURE 4:**
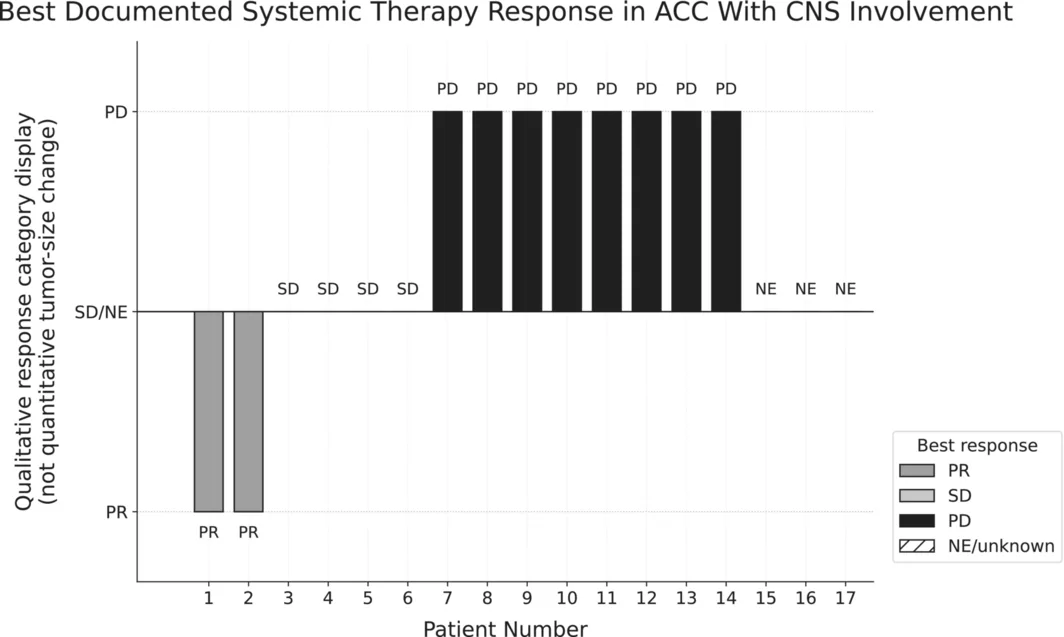
Best documented systemic therapy response by patient. Qualitative summary of best documented systemic therapy response among patients receiving treatment after CNS involvement. Response categories were abstracted retrospectively from treating oncology documentation and included partial response (PR), stable disease (SD), progressive disease (PD), and non‐evaluable or unknown response. Because formal lesion‐level radiographic measurements were not uniformly available, this figure should not be interpreted as a formal waterfall plot of measured percentage change in tumor burden.

### 3.4 Survival Outcomes

Median progression‐free survival after CNS involvement was 5.4 months, with a 95% confidence interval of 1.8–10.5 months. Median overall survival after CNS involvement was 9.4 months, with a 95% confidence interval of 3.2–20.8 months. Median duration of follow‐up from CNS diagnosis was 7.9 months (Figure 2).

Survival appeared to vary by clinical subgroup, particularly according to ECOG performance status and leptomeningeal involvement. Patients with leptomeningeal disease had especially poor outcomes, with no affected patients alive at the time of data cutoff. By contrast, several patients without leptomeningeal disease were alive at last follow‐up. Similarly, no patients with NOTCH1 alterations were alive at the time of data cutoff, although this observation should be interpreted cautiously because of the small sample size and potential confounding by disease burden, treatment exposure, and CNS disease pattern.

These subgroup findings are descriptive and hypothesis‐generating. The cohort was not powered to determine whether ECOG status, leptomeningeal involvement, NOTCH1 alteration status, or CNS metastatic pattern independently predicted survival.

## 4 Discussion

CNS involvement in ACC is uncommon and remains poorly characterized [15, 16]. In this single‐institution cohort of 17 patients, CNS disease was associated with short progression‐free and overall survival despite multimodal therapy. Median progression‐free survival was 5.4 months, and median overall survival was 9.4 months after CNS involvement. These findings support the clinical impression that CNS involvement represents a high‐risk event in ACC, although the small sample size and retrospective design preclude definitive prognostic modeling.

A major finding of this study is the heterogeneity of CNS involvement. CNS disease in ACC should not be considered a single entity. Some patients had discrete parenchymal brain lesions suggestive of hematogenous spread, whereas others had skull base, cranial nerve, dural, brainstem‐adjacent, or leptomeningeal patterns suggestive of neurotropic or perineural extension. ACC is particularly notable among head and neck malignancies for frequent perineural invasion, and perineural spread across the skull base may involve the trigeminal nerve, facial nerve, cavernous sinus, Meckel cave, skull base foramina, dura, or adjacent intracranial structures [4, 17, 18]. This distinction is clinically important. Predominantly parenchymal lesions may be amenable to focal therapies such as resection or stereotactic radiosurgery, whereas skull base/perineural patterns and leptomeningeal disease often involve anatomically complex regions not amenable to local therapies and may produce cranial neuropathies, brainstem compression, or diffuse neurologic morbidity.

Leptomeningeal involvement was present in 6 patients and appeared to be associated with particularly poor outcomes. This likely contributed substantially to the poor aggregate survival observed in the cohort. For this reason, survival estimates from CNS‐metastatic ACC cohorts should be interpreted in the context of disease pattern. Predominantly parenchymal lesions, skull base/perineural involvement, and leptomeningeal dissemination may represent clinically and biologically distinct states rather than interchangeable manifestations of the same process.

The observed frequency of NOTCH1 alterations is notable but should be interpreted cautiously. NOTCH1 alterations were identified in 7 of 17 patients, a proportion numerically higher than that reported in several published ACC cohorts [19, 20, 21, 22, 23, 24]. However, this study did not include a matched non‐CNS ACC control group, and comparisons with prior literature are indirect. We therefore cannot conclude that NOTCH1 alterations are definitively enriched in CNS‐metastatic ACC. Instead, these findings suggest that NOTCH1 alterations may be overrepresented in this clinically aggressive cohort and warrant further study in larger multi‐institutional datasets with matched CNS‐negative comparators.

The biological plausibility of this observation is supported by prior studies identifying NOTCH1‐mutant ACC as an aggressive molecular subgroup. Activating NOTCH1 alterations have been associated with advanced‐stage disease, solid histology, early relapse, distant metastatic spread, and inferior survival [21, 24]. Whether NOTCH pathway activation specifically contributes to CNS tropism, perineural invasion, or leptomeningeal dissemination, however, remains uncertain. Nevertheless, the overlap between NOTCH1‐mutant disease, aggressive metastatic behavior, and the intrinsic neurotropic biology of ACC provides a rationale for further investigation. Future studies should integrate genomic profiling with detailed radiographic annotation of perineural spread, skull base involvement, and hematogenous parenchymal metastases.

Systemic therapy outcomes were poor, but response interpretation is limited by retrospective qualitative response documentation and incomplete lesion‐level measurements. ACC is generally resistant to cytotoxic chemotherapy, and systemic agents have limited CNS penetration [8, 9, 12, 13]. In this cohort, objective responses were uncommon, with only 2 patients having a documented partial response and no complete responses. However, because baseline and nadir target‐lesion measurements were not uniformly available, formal lesion‐level radiographic response assessment could not be performed across the full cohort. In addition, intracranial and extracranial responses could not be reliably distinguished for every patient. Figure 4 should therefore be interpreted as a qualitative response‐category summary rather than a formal waterfall plot of measured percentage change in tumor burden. Future studies should prospectively collect lesion‐level measurements and report intracranial and extracranial responses separately.

Our findings have practical implications for clinical care and future research. Patients with ACC and CNS involvement should undergo careful radiographic assessment to distinguish predominantly parenchymal disease from skull base/perineural involvement and leptomeningeal disease [17, 18]. Treatment decisions should account for performance status, neurologic symptom burden, extracranial disease control, feasibility of focal CNS‐directed therapy, and expected systemic therapy benefit. Molecular profiling should be considered when tissue is available as it may inform clinical trial eligibility and future development of improved systemic therapies. Given the poor prognosis observed in patients with leptomeningeal involvement, early integration of palliative care and proactive goals‐of‐care discussions are appropriate.

This study has several limitations. The cohort is small, retrospective, and derived from a single institution. The absence of a matched ACC cohort without CNS involvement prevents definitive conclusions about genomic enrichment or CNS‐specific risk factors. CNS involvement patterns were heterogeneous, and some cases could not be confidently assigned to a single route of CNS involvement. NGS testing platforms varied across patients. Systemic therapy exposures were heterogeneous, and retrospective response assessment was limited by nonuniform imaging intervals and incomplete lesion‐level measurements. Accordingly, all statistical and genomic associations should be considered exploratory and hypothesis‐generating.

In conclusion, CNS involvement in ACC is associated with poor outcomes and includes biologically heterogeneous patterns of disease, including predominantly parenchymal lesions, skull base/perineural involvement, and leptomeningeal dissemination. NOTCH1 alterations and leptomeningeal disease were frequent in this cohort, but their prognostic and biologic significance requires validation. Multi‐institutional studies with matched CNS‐negative controls, standardized imaging review, lesion‐level response assessment, and integrated genomic analysis are needed to define risk factors for CNS involvement and identify effective CNS‐penetrant therapeutic strategies.

## Funding

This study did not require financial support from any institutions or third‐party sources.

## Ethics Statement

This study was approved by the University of Pennsylvania Institutional Review Board (approval number #857499).

## Consent

The authors have nothing to report.

## Conflicts of Interest

The authors declare no conflicts of interest.

## Supporting information


**Figure S1:** Binary heatmap of additional pathogenic or likely pathogenic alterations. Expanded heatmap showing less frequent molecular alterations grouped under Other in Figure 1.

## Data Availability

The data that support the findings of this study are available on request from the corresponding author. The data are not publicly available due to privacy or ethical restrictions.
